# Finite Element Analysis of an Implant-Supported FDP with Different Connector Heights

**DOI:** 10.3390/sym14112334

**Published:** 2022-11-07

**Authors:** Laura H. J. Alberto, Lohitha Kalluri, Josephine F. Esquivel-Upshaw, Yuanyuan Duan

**Affiliations:** 1 Department of Biomedical Materials Science, University of Mississippi Medical Center, Jackson, MS 39216, USA; 2 Department of Restorative Dental Sciences, Division of Prosthodontics, University of Florida, Gainesville, FL 32611, USA

**Keywords:** all-ceramic FDP, connector design, connector height, dental biomaterials, dental implants, design parameters, finite element analysis

## Abstract

All-ceramic fixed dental prostheses (FDPs) tend to fracture in the connector areas, due to the concentration of tensile stresses. This study aimed to evaluate the role of connector height on the stress distribution of a posterior three-unit implant-supported all-ceramic FDP using finite element analysis (FEA). Two titanium dental implants, their abutments, screws, and a three-unit all-ceramic FDP were scanned using a micro-CT scanner. Three 3D models with altered distal connector heights (3, 4, and 5 mm) were generated and analyzed on ABAQUS FEA software. The maximum principal stress values in MPa observed for each model with different connector heights and their respective locations (MA = mesial abutment; DA = distal abutment; F = framework; V = veneer) were: 3 mm—219 (MA), 88 (DA), 11 (F), 16 (V); 4 mm—194 (MA), 82 (DA), 8 (F), 18 (V); 5 mm—194 (MA), 80 (DA), 8 (F), and 18 (V). All the assembled models demonstrated the peak stresses at the neck area on the mesial abutments. The connector height had a significant influence on the stress distribution of the prosthesis. The models with higher distal connectors (4 and 5 mm) had a lower and more uniform distribution of maximum principal stresses (except for the veneer layer) when compared with the model with the smallest distal connector.

## 1. Introduction

The increasing demand for highly aesthetic and metal-free prostheses has made all-ceramic fixed dental prostheses (FDPs) a popular choice among dentists and patients in dental rehabilitation [1–5]. Ceramics are well known for their ability to mimic the tooth’s optical characteristics, great biocompatibility, and chemical stability [4,6,7]. In addition, the development of ceramics, such as yttria-stabilized tetragonal zirconia polycrystal (3Y-TZP) with high flexural strength (800–1500 MPa) [8] and fracture toughness (5.5–7.4 MPa·m^1/2^) [9], has made it possible to substitute traditional metal frameworks with ceramic frameworks on FDPs. Survival rates for these all-ceramic restorations are 90.4% after 5 years [10] and 91.3% after 10 years [11] even in posterior sites [1,5,10,12]. Zirconia has also been used as an implant abutment material in anterior and posterior regions, with a reported three-year survival rate of 100% [13]. Even though zirconia presents with excellent mechanical properties (due to the tetragonal to monoclinic transformation), most dental zirconia are opaque and need to be veneered, usually with lithium disilicate glass-ceramic, to reproduce the natural aesthetics of teeth [4,14–16].

Computer-aided design/computer-aided manufacturing (CAD/CAM) technology has been successfully used to produce complex shapes of all-ceramic FDP prostheses using medical imaging data. In this process, both the zirconia framework and veneer are fabricated by CAD/CAM and combined by fusion. This technique eliminates flaws and consequently strengthens the veneer layer, generating high-strength bilayer ceramics when compared with other techniques, such as overpressed or hand-layered veneers [17–19]. Therefore, the use of CAD/CAM reduces the incidence of chipping, which has been identified as a major technical complication for veneered all-ceramic FDPs [9,20].

Implant-supported dental restorations have been the treatment of choice to rehabilitate partial edentulism due to their high success rates, the preservation of adjacent teeth, and ability to support high mastication forces [21,22]. A meta-analysis demonstrated an estimated five-year survival rate of 93% after analyzing 175 implant-supported zirconia-ceramic FDPs [23]. An in vitro mechanical test with three-unit implant-supported zirconia-based FDPs reported fracture load values of 1789 ± 202 N for axial and 1200 ± 68 N for oblique loads [24].

Many factors, such as the position in the arch, number of units, material composition, fabrication technique, and design of the prosthesis, have been cited as factors that may affect the longevity of all-ceramic FDPs [25]. Among them, the prosthesis design has been proven to be one of the most relevant factors. The fracture of all-ceramic FDPs tends to occur in the connector area, starting from the gingival embrasure and propagating until the occlusal surface [25–31]. The explanation for such a phenomenon is that all-ceramic implant-supported FDP under masticatory forces behaves like a ceramic beam on rigid supports [8]. During the mastication, compressive forces are applied on the occlusal surface of the prosthesis. Therefore, the narrow constrictions with asymmetric irregular shapes on the connectors’ area concentrate tensile stress. As a brittle material, ceramics tend to fail in tension by a lack of ductility [29]. Based on that, and following the theory of the deflection of a beam (where the height cubed is inversely proportional to the deflection), it was assumed that the higher the load on the FDP, the higher the connector height has to be [26]. However, posterior regions present limited space and the highest masticatory forces (up to 500 N in an average person) [24]. Hence, the distal connectors of posterior prostheses have a higher incidence of fractures when compared with the mesial connectors [25,26]. Thus, in this study, we altered the distal connector height of a three-unit implant-supported FDP to assess the stress distribution within the all-ceramic prosthesis as well as the mesial and distal zirconia abutments.

Load-to-fracture in vitro testing, such as static load-bearing tests, is a common way to assess the mechanical strength of FDPs [32]. The most common techniques are three or four-point bend tests. Although these techniques are consolidated to test all-ceramic FDPs, they usually require a large number of specimens (to overcome the flaw variation) and controlled loading conditions, making these tests costly and technique-sensitive. Another drawback of in vitro tests is that they exclusively provide the final fracture load value and lack data about how the distribution of stresses happened until the fracture occurred [8,25]. In trying to solve these limitations as well as to provide a better understanding of experimental tests and improve the predictability of rehabilitation, finite element analysis (FEA) was introduced in Dentistry [33]. The FEA enables the calculation and assessment of the stress distribution in each part of an asymmetric complex structure, such as an FDP. Additionally, the selection of different failure criteria depending on the material nature is possible. For structures made of brittle materials, such as all-ceramic FDPs, the maximum principal stress is the failure criterion of choice for representing the magnitude of tensile stress. Thus, maximum principal stress values were considered for evaluation in this study. The acquisition of high-accuracy models for further analysis can be carried out by multiple methods, such as three-dimensional (3D) scanners, computer tomography, or the measurement of physical specimens. Furthermore, the specific parameters of models, such as geometry, design, material properties, and loading direction, as well as load intensity can be modified to create new desired models for analyses. For all these reasons, FEA has proven to be a powerful tool to test models in a reliable, relatively fast, economically reasonable, and non-destructive way [34]. Thus, 3D FEA was carried out to study the stress distribution in this study.

In the current study, the distal connector of a clinically retrieved posterior three-unit all-ceramic FDP produced by CAD/CAM technique was modified. Three different models with distal connector heights measuring 3, 4, and 5 mm have been created and analyzed by FEA. The objective of this study was to assess the impact of the distal connector design on the stress distribution and biomechanical behavior of fracture in posterior implant-supported three-unit all-ceramic FDPs. Many FEA studies have been performed on all-ceramic FDP. However, they used 2D models [27,35] or focused on different scenarios involving FDP, such as four-unit prostheses [36], distal cantilevers [37], different connector designs [38], or rehabilitation with inlay restorations [39,40]. This study was the final part of a series of studies [41,42] that aimed to evaluate different design parameters that affect the failure of ceramic–ceramic prostheses based on clinical observations from previous publications [43,44]. The null hypothesis was that the distal connector with the highest connector height (5 mm) would present the lowest maximum principal stress (measurement of tensile stresses) on the gingival aspect of the connectors on the veneer and framework layers as well as in mesial and distal ceramic abutments, promoting a more uniform and beneficial stress distribution throughout the prosthesis and adjacent components.

## 2. Materials and Methods

### 2.1. Geometry Acquisition

**Figure 1.**
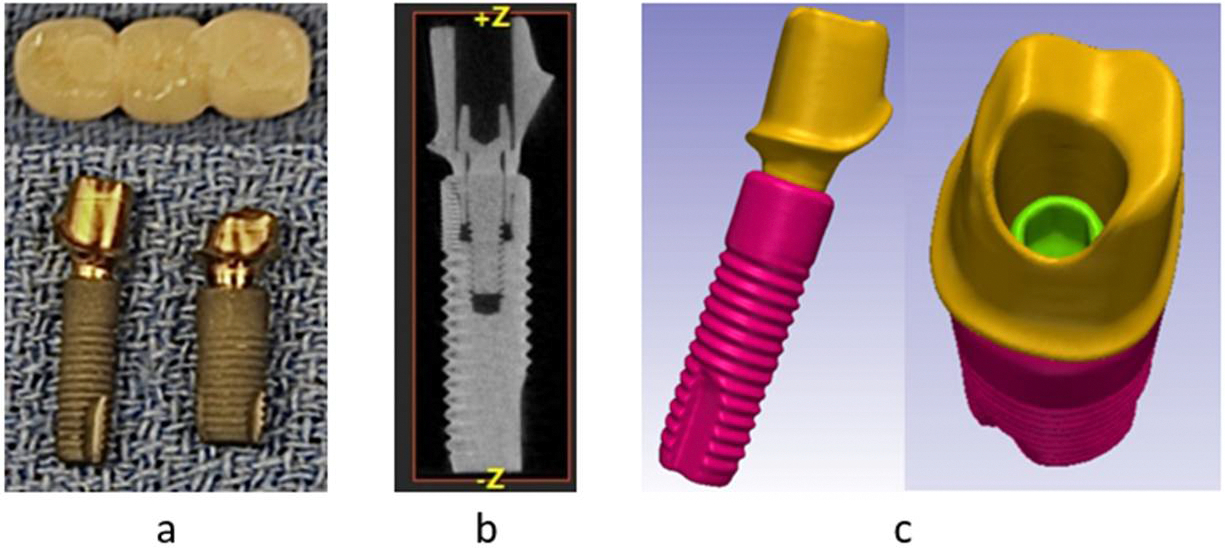
(**a**) Physical specimens, (**b**) the reconstructed micro-CT image of the Ø 4.0 implant with its respective abutment and screw, and (**c**) the 3D model of the same implant.

For this study, two implants with 4.0 and 4.8 mm diameters (Astra Tech Osseospeed, Dentsply Sirona, York, PA, USA), their suitable abutments and screws were all scanned in a micro-CT scanner (Skyscan1172; Bruker, Aartselaar, Belgium). The same technique was used to make the geometry acquisition of a patient-retrieved three-unit all-ceramic FDP produced by CAD/CAM technique (Figure 1a). The resolution of micro-Ct scanning was 34.4 μm and the additional parameters were 100 μA for the accelerating current as well as 100 kV for voltage. The sliced images generated from each physical component cited previously were reconstructed using a micro-CT reconstruction software (Skyscan NRecon software; Bruker, Aartselaar, Belgium).

The reconstructed micro-CT images were imported into an interactive medical image processing software (Simpleware ScanIP; Synopsys, Mountain View, CA, USA) to be developed into 3D models (Figure 1b,c). Individual masks of all components were produced using thresholding tools based on their greyscale values of image pixels. Other tools, such as 3D editing, Boolean operations, and recursive Gaussian filters, were used to correct artifacts and imperfections. Geometrical forms that simulated bone and the fillings inside the abutments were generated with the Create object tool while the cement layers between the abutments and their respective implants were generated using morphological close and Boolean tools.

**Figure 2.**
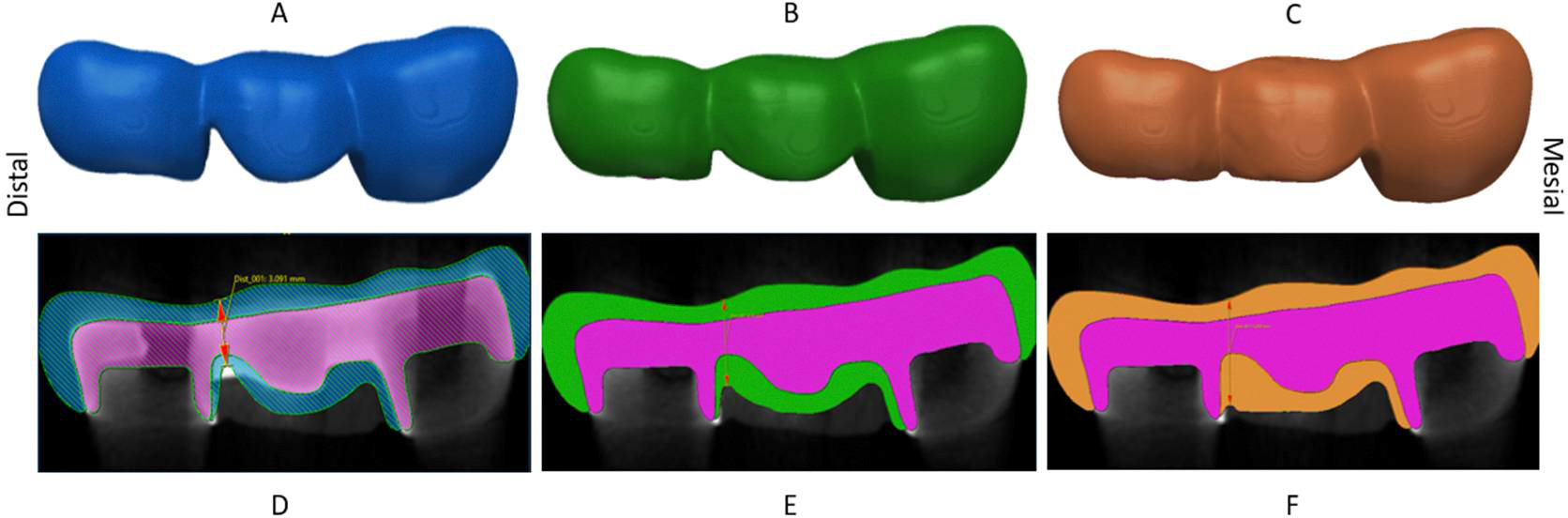
On the top row, the buccal view of (**A**) model A with 3 mm, (**B**) model B with 4 mm, and (**C**) model C with 5 mm connector height. On the bottom row, sectional images of (**D**) model A, (**E**) model B, and (**F**) model C.

The distal connector height of the bilayer ceramic prosthesis was altered to create three distinct 3D models. Model A presented a distal connector with a height of 3 mm, followed by model B with 4 mm, and model C with 5 mm, as depicted in Figure 2. The modification was carried out exclusively on the gingival aspect of the veneer layer mask, keeping the same features on the remaining masks. A 0.25 mm radius of the curvature of gingival embrasure was used in the distal connector of the three models, along with the occlusal veneer thickness of 0.9 mm, the zirconia framework vertical thickness of 1.5 mm, and the connector width of 4 mm. The mesial connector presented approximately 4.5 mm connector height and 5 mm width in models A, B, and C.

**Figure 3.**
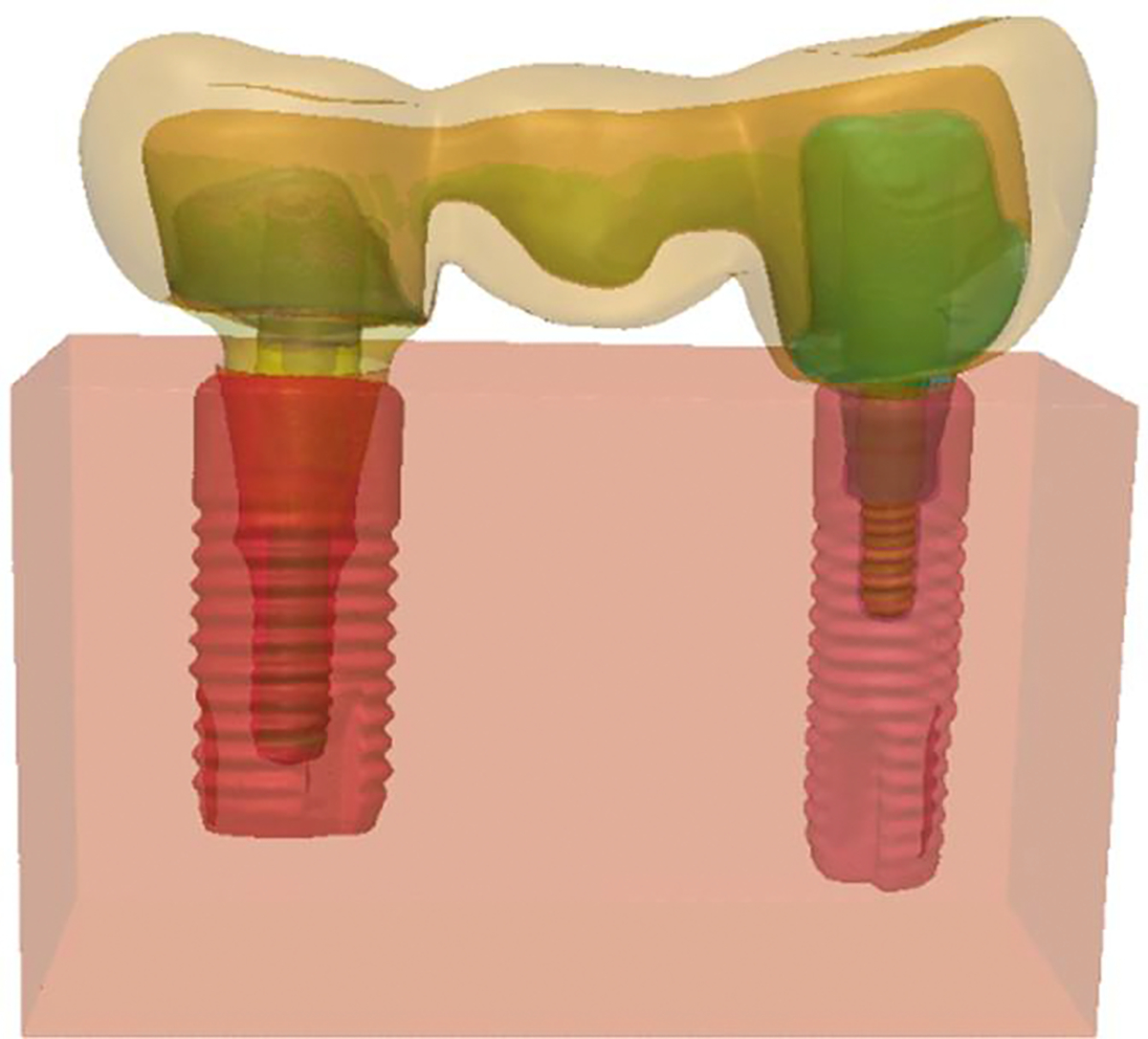
Model B assembled simulating the original anatomic position of the components.

All masks were converted into surfaces by Standard Tessellation Language (STL) files to describe the surface geometry of the three-dimensional objects and imported to a new Simpleware file to be assembled simulating their original anatomic positions, as illustrated in Figure 3.

### 2.2. Pre-Processing

All models were meshed using tetrahedral elements, the most commonly used element type for biomedical FEA studies [45]. The INP files generated after each meshing were analyzed using commercial finite element analysis software (ABAQUS; Dassault Systèmes Simulia Corp., Johnston, RI, USA). All the material’s properties were considered to be homogeneous, linear elastic, and isotropic for model simplification, even though the materials can present a more complex behavior [34]. Elastic material properties used for each specific component of the models were summarized in Table 1 [41,42,46]:

All the simulated bone external surface nodes were fixed (configuring a boundary condition) to prevent displacement and rotation of the model and simulate the anatomical conditions. A 110 N vertical load was applied at the occlusal surface of the pontic at the central fossa of models A, B, and C to mimic a mechanical three-point bending test. The load was applied to 500 nodes and equally divided, generating a load of 0.22 N/node.

### 2.3. Post-Processing

After running a full FEA of the models, the maximum principal stress data were collected. This stress type was chosen due to the brittle nature of ceramics and the fact that they usually fail when submitted to concentrated tensile stress. The stress distribution and peak stresses from the assembled mesh volumes and their ceramic veneer, framework, and abutments layers were recorded and analyzed.

## 3. Results

**Figure 4.**
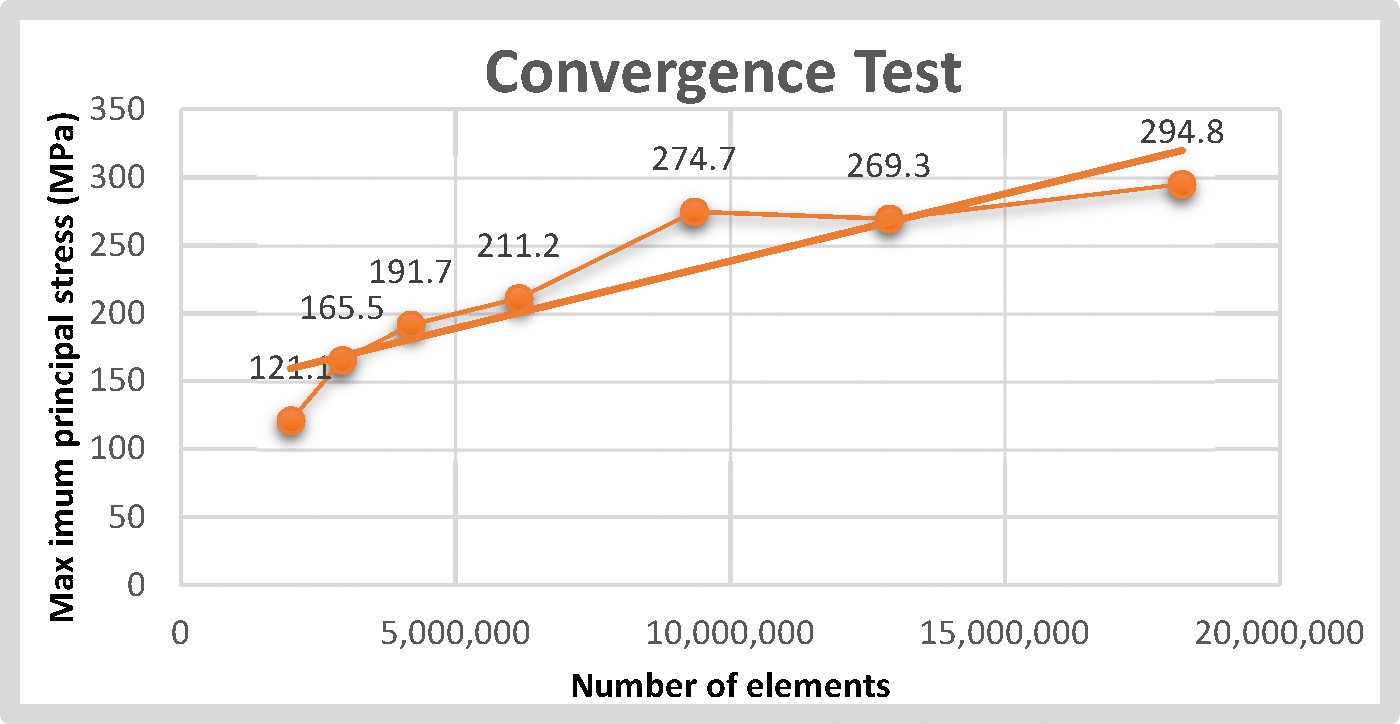
Convergence test data.

The convergence test was conducted using seven different mesh densities and their respective results. A graphic was plotted correlating the number of elements for each mesh density and the corresponding results of the test expressed in maximum principal stress, as represented in Figure 4. The mesh density that was chosen as the most appropriate to be applied in the remaining models was the −20 mesh density with a peak stress of 274.7 MPa. Among all the mesh densities tested, the chosen mesh density ensured that the results of the analysis were not affected by the refinement of the mesh (because they were converging to a repeatable solution on the subsequent mesh densities) and provided the smallest solving time. The total number of elements for each assembled model with this optimized mesh density was 9,308,115 for model A, 9,317,208 for model B, and 9,317,088 for model C, respectively.

**Figure 5.**
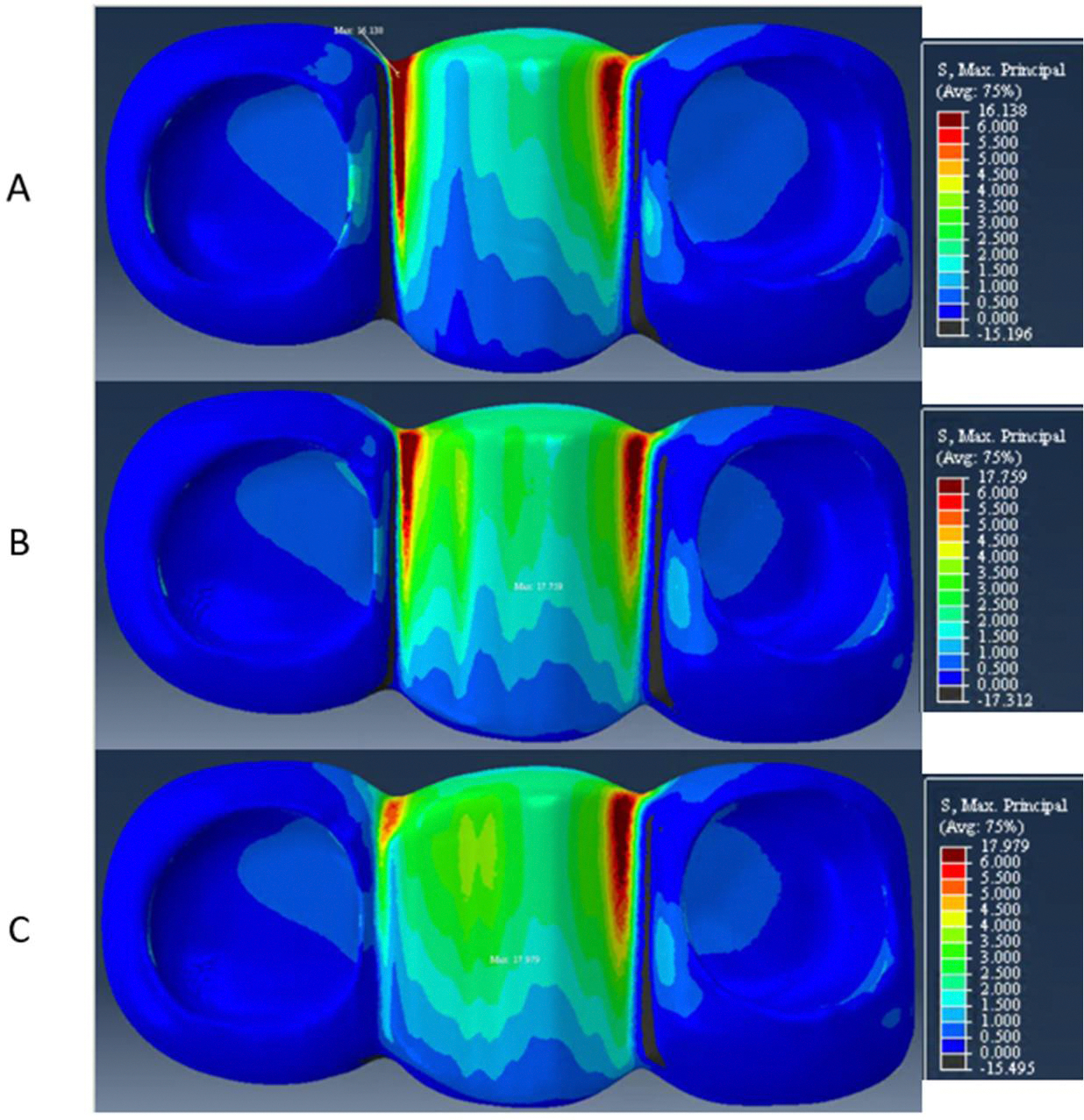
Stress distribution on veneer for (**A**) model A, (**B**) model B, and (**C**) model C. Note the concentration of tensile stresses on the distal connector on models A and B (3 and 4 mm) and the anterior connector on model C (5 mm).

The peak stresses in the veneer layer were located at the gingival aspect of the distal connector in model A and at the occlusal surface of the pontic, near the loading area, in models B and C. The veneer peak stress values were 16 MPa for model A, 18 MPa for model B, and 18 MPa for model C. When considering just the gingival aspect of the veneer layer, the peak stresses within this specific area were 16 MPa at the distal connector region for model A, 10 MPa at the distal connector region for model B, and 8 MPa at the mesial connector region for model C, as represented in Figure 5. Note that in the contour plots used in the current study the hotter colors represent the higher maximum principal stresses and the colder colors represent the lower maximum principal stresses.

**Figure 6.**
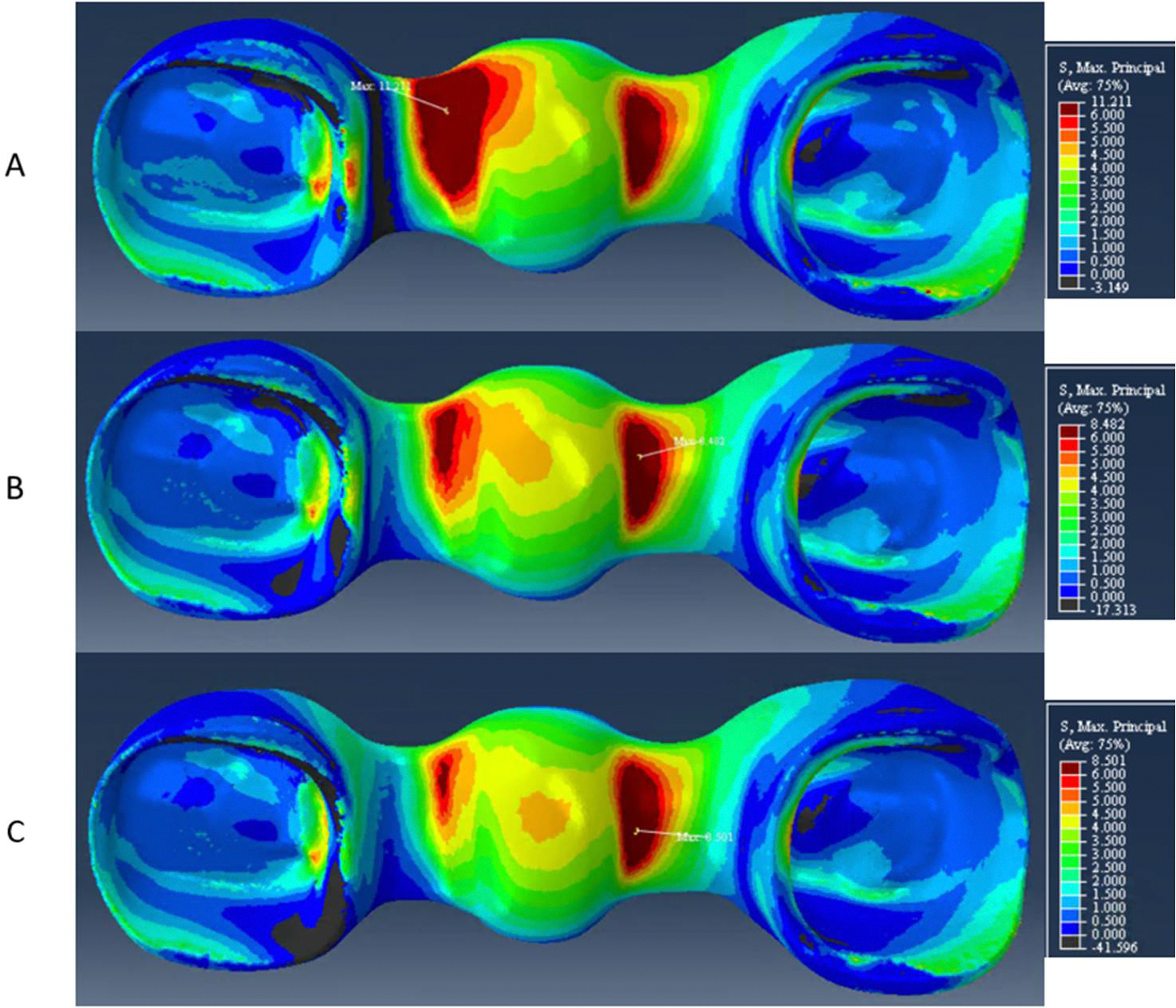
Maximum principal stresses contours on the zirconia framework for (**A**) model A, (**B**) model B, and (**C**) model C. Observe the shifting of peak stresses from the distal connector on model A (**A**) to the mesial connector on models B and C (**B**,**C**).

The zirconia frameworks presented high-stress values at the gingival aspect of the distal connector for model A, while in models B and C they were concentrated at the gingival aspect of the anterior connector. Maximum principal stresses were 11, 8, and 8 MPa respectively, in models A, B, and C, as shown in Figure 6.

**Figure 7.**
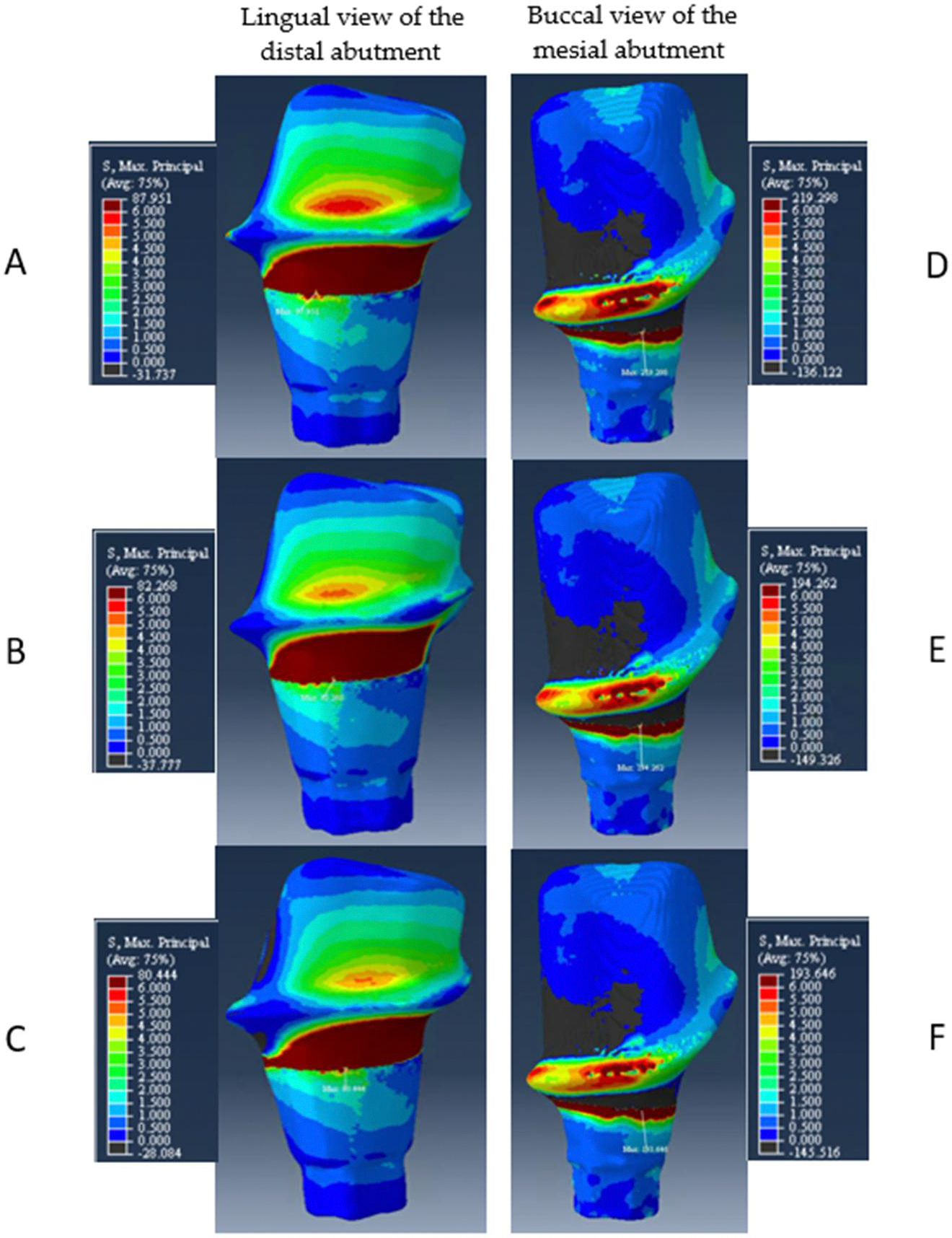
Distribution of maximum principal stress. On the left column, the lingual views of the distal abutments are depicted for (**A**) model A, (**B**) model B, and (**C**) model C. On the right column, the contour plots for the buccal view of the mesial abutments are depicted for (**D**) model A, (**E**) model B, and (**F**) model C.

All the assembled models demonstrated the peak stresses at the neck area on the buccal face of the mesial abutment. Model A, with a 3 mm connector height, presented the highest peak stress value (219 MPa) when compared with the other models B (194 MPa) and C (194 MPa). The peak stresses on the distal abutment were also located in the neck area but at the lingual face. The maximum principal stress for models A, B, and C were 88, 82, and 80 MPa, respectively. The distribution of stresses on the mesial and distal abutments is demonstrated in Figure 7.

A summary of the peak stresses in the individual layers of models A, B, and C is shown in Table 2. All values are representing measurements of maximum principal stress.

## 4. Discussion

The FEA method was used in several studies [21,27,47,48] that have investigated the influence of the connector design, especially the connector height. All of them assessed the stress distribution on all-ceramic three-unit posterior FDPs. Because the connectors represent the thinnest cross-section of an FDP, this area is more susceptible to stress concentration. The results of these studies were in accordance with the results of the current study, as well as the null hypothesis. As expected, increasing the connector height also increases the connector cross-section area where the stresses are concentrated, therefore promoting a more uniform stress distribution and lower peak stresses. Bataineh [47] and coworkers investigated posterior all-ceramic three-unit FDP connectors with 4 × 3 mm; 3.75 × 2.75 mm; and 3.5 × 2.5 mm sizes. They concluded that the connector size plays a key role in the long-term survival of the prostheses. Their results showed that, by reducing the connector size, a significant reduction in fatigue strength could be obtained, with values going from 670 N in the largest connector (4 × 3 mm) to 273 N on the smallest connector (3.5 × 2.5 mm). In their study, all the failures occurred on distal connectors. In another study [27], after testing FDP models with 3 and 4 mm connector heights, the authors observed that the stresses were concentrated in the connector regions. They also concluded that an increase in the connector height would dramatically reduce the stress levels within the connectors. The presence of tensile peak stress near the occlusal contact point (as shown in models B and C of the current study) was also found in a recent FEA study [49] of all-ceramic three-unit posterior FDPs. However, the load applied was 300 N, but the measurements of the connectors used were not mentioned. The authors found maximum principal stress values ranging from 17.5 to 37.4 MPa for the connector areas and from 10.8 to 22.0 MPa on the gingival aspect of the zirconia framework.

The relationship between zirconia framework and implant-supported prostheses located on posterior mandibular sites has also been assessed. In an FEA study [50] frameworks made of six materials (pure titanium, cobalt-chromium alloy, gold alloy, zirconia, polyether ether ketone (PEEK), and carbon fiber-reinforced PEEK) were analyzed under a 300 N load with a 75° inclination. The results showed that the highest stresses were located around the neck of the implants and cortical bone. They conclude that the more beneficial stress distribution occurred in prostheses with zirconia and metal framework. Vult von Steyern et al. [51] drew a similar conclusion when comparing all-ceramic FPD supported by implants and by tooth-analog abutments in an in vitro study. The prostheses supported by implants had statistically significant higher loads at the fracture, leading the authors to conclude that prostheses supported by implants were able to provide more advantageous stress distribution than the ones supported by tooth analogs. Both studies as well as the current study proved that prostheses with zirconia frameworks supported by the implants (as used in the current study), were able to present good stress distribution when compared with other alternatives.

Physical specimens were also tested in vitro to evaluate the impact of connector height in all-ceramic posterior FDPs [26,52,53]. After testing forty-eight three-unit all-ceramic FPDs, with connector height and width ranging from 2 to 4 mm, Bahat et al. [52] recommended minimal dimensions of 3 mm in height and 2 mm in width for this type of prostheses. Another in vitro study [53] tested seventy three-unit FDPs with connector height and width measuring 3 mm × 3 mm, 3.5 mm × 3.5 mm, and 4 mm × 4 mm, respectively, and a radius of the gingival embrasure of 0.6 mm. They concluded that the fracture resistance of the zirconia framework significantly increased as the connector diameters increased. The in vitro studies cited fabricated their specimens with a symmetric design, which is not always possible in the clinical scenario. Posterior FDPs have asymmetric shapes (due to limited space in posterior regions in the mouth and aesthetics) and support the highest masticatory forces during the chewing process [25,26], which influences the distribution of stresses within the prosthesis components, as shown in the current study.

Regarding the clinical studies, few studies [43,44,54] have been carried out to analyze the effects of design parameters, including connector height. Sixty-five implant-supported zirconia-ceramic FDPs with connector heights of 3, 4, and 5 mm were evaluated in a randomized controlled clinical trial [44]. The presence of cracks and fractures was assessed in a five-year follow-up study. A total of sixteen prostheses presented fractures, including seven fractures in the 3 mm group, six fractures in the 4 mm group, and three fractures in the 5 mm height group. The fact that the higher number of fractures was found in the group with the smallest connector can be explained by the higher peak stress observed in the same group in the current study. Only chipping fractures were presented, with no occurrences of fractures on the connector. This phenomenon also corroborates with the FEA results in this study, as the peak stresses found on the gingival aspect of veneer and framework were much lower than the flexural strength of the zirconia (800–1500 MPa) [8], making a fracture at the connector areas unlikely. Although the results showed more fractures in the 3 mm group, statistical survival analysis did not demonstrate a statistically significant effect between the connector height (*p* = 0.89) and fracture. Clinically, framework fractures are rare on zirconia-ceramic FDPs [55]. A systematic review [56] showed that less than 1% of these prostheses (5 of 595 FDPs) had the zirconia framework fracture.

This study was designed to simulate clinical scenarios where implants were used as support. The presence of implants with different diameters (Ø 4.0 and 4.8 mm) placed with a slight angulation is a common occurrence in clinical rehabilitations. The use of these strategies is often necessary to adapt to restricted anatomical sites and high loads on posterior areas. All these factors, as well as the modification of the connector height on the distal connector of the models, can influence the stress distribution in all the components of the prosthesis and possibly cause high-stress concentrations on the neck region of the mesial and distal zirconia abutments. Model A, with 3 mm posterior connector height, presented higher peak stresses when compared with models B and C. It should be noted that the high values on the mesial zirconia abutment are less likely to cause the failure of this component due to the high strength of this material. A literature review [57] showed that zirconia abutments had a similar survival rate when compared to titanium abutments (100%) after a follow-up of 11 years. Additionally, there were no significant differences in biological and radiographic indexes among metals, ceramics, and natural teeth abutments.

This study showed that FEA is an excellent tool to analyze the stress distribution and locations of stress concentration in a three-unit all-ceramic implant-supported FDP with different connector heights. Future studies can be carried out by simulating other clinical scenarios, material properties, prosthetic designs, and loading conditions. It should be noted that, although with high dimensional accuracy and reliable results, FEA studies present limitations. As the limitations of this study, all the material properties were considered homogeneous, linear elastic, and isotropic and the models were simplified to achieve reduced run time and more efficient analysis, while in the reality, the material properties followed a complex pattern as well as the features of the models’ components. Additionally, other clinical factors, such as antagonistic teeth, masticatory frequency, and combination of different load directions, were not simulated in this study to better control model complexity.

## 5. Conclusions

Finite element analysis results demonstrated that the connector height has a significant influence on the stress distribution on implant-supported FDPs. Models with higher distal connectors (5 and 4 mm) had a lower and a more uniform distribution of maximum principal stresses (except for the veneer layer) when compared with the model with the shortest distal connector (3 mm). The maximum principal stress values in MPa observed for each model with different connector heights and their respective locations (MA = mesial abutment; DA = distal abutment; F = framework; V = veneer) were: 3 mm—219 (MA), 88 (DA), 11 (F), 16 (V); 4 mm—194 (MA), 82 (DA), 8(F), 18 (V); 5 mm—194 (MA), 80 (DA), 8 (F), and 18 (V). The peak tensile stresses in the model with the shortest distal connector were located at the modified connector on the veneer and framework, while in the other two models with wider connectors the peak stresses shifted to the mesial connector. Special attention should be paid to the neck area of the zirconia abutments due to the high-stress values observed among all the models analyzed.

## Figures and Tables

**Table 1.** Material properties of all components of the mesh volumes [41,42,46],

Materials	Model Components	Young’s Modulus (MPa)	Poisson’s Ratio
Porcelain	Veneer	70,000	0.19
Zirconia	Framework Abutments	210,000	0.30
Resin cement	Fillings Cement layer	8300	0.30
Titanium	Implants Screws	110,000	0.35
Bone	Cuboid (bene)	13,700	0.30

**Table 2.** Summary of maximum principal peak stress values on the layers of models A, B, and C.

Models	Distal Connector Height	Veneer	Veneer (Gingival Aspect)	Framework	Mesial Abutment	Distal Abutment
A	3 mm	16 MPa	16 MPa	11 MPa	219 MPa	88 MPa
B	4 mm	18 MPa	10 MPa	8 MPa	194 MPa	82 MPa
C	5 mm	18 MPa	8 MPa	8 MPa	194 MPa	80 MPa

## Data Availability

Not applicable.
